# Multidimensional outcome assessment of pulmonary rehabilitation in traits-based clusters of COPD patients

**DOI:** 10.1371/journal.pone.0263657

**Published:** 2022-02-17

**Authors:** Ingrid M. L. Augustin, Frits M. E. Franssen, Sarah Houben-Wilke, Daisy J. A. Janssen, Swetlana Gaffron, Herman-Jan Pennings, Frank W. J. M. Smeenk, Willem R. Pieters, Amber Hoogerwerf, Arent-Jan Michels, Frits van Merode, Emiel F. M. Wouters, Martijn A. Spruit

**Affiliations:** 1 Ciro, Center of Expertise for Chronic Organ Failure, Horn, The Netherlands; 2 NUTRIM School of Nutrition and Translational Research in Metabolism, Maastricht University Medical Centre+, Maastricht, The Netherlands; 3 Department of Respiratory Medicine, Maastricht University Medical Centre+, Maastricht, The Netherlands; 4 Department of Health Services Research, Care and Public Health Research Institute, Faculty of Health, Medicine and Life Sciences, Maastricht University Medical Centre+, Maastricht, The Netherlands; 5 Viscovery Software GmbH, Vienna, Austria; 6 Department of Respiratory Medicine, Laurentius Hospital, Roermond, The Netherlands; 7 Department of Respiratory Medicine, Catharina Hospital, Eindhoven, The Netherlands; 8 Department of Respiratory Medicine, Elkerliek Hospital, Helmond, The Netherlands; 9 Department of Respiratory Medicine, St. Jans Gasthuis, Weert, The Netherlands; 10 Department of Respiratory Medicine, St. Anna Hospital, Geldrop, The Netherlands; 11 School for Public Health and Primary Care, Faculty of Health, Medicine and Life Sciences, Maastricht University Medical Centre+, Maastricht, The Netherlands; 12 Ludwig Boltzmann Institute for Lung Health, Vienna, Austria; Universite de Toulon, FRANCE

## Abstract

**Background:**

Clusters of COPD patients have been reported in order to individualize the treatment program. Neither co-morbidity clusters, nor integrated respiratory physiomics clusters contributed to a better prediction of outcomes. Based on a thoroughly assessed set of pulmonary and extra-pulmonary traits at the start of a pulmonary rehabilitation (PR) program, we recently described seven clusters of COPD patients. The aims of this study are to confirm multidimensional differential response and to assess the potential of pulmonary and extra-pulmonary traits-based clusters to predict this multidimensional response to PR pulmonary in COPD patients.

**Methods:**

Outcomes of a 40-session PR program for COPD patients, referred by a chest physician, were evaluated based on the minimal clinically important difference (MCID) for 6-minute walk distance (6MWD), cycle endurance time, Canadian Occupational Performance Measure performance and satisfaction scores, Hospital Anxiety and Depression Scale anxiety and depression scores, MRC dyspnea grade and St George’s Respiratory Questionnaire. The aforementioned response indicators were used to calculate the overall multidimensional response and patients were grouped in very good, good, moderate and poor responders. In the same way, responses to pulmonary rehabilitation were compared based on seven previously identified pulmonary and extra-pulmonary traits-based clusters.

**Results:**

Of the whole sample, drop out was 19% and 419 patients (55.4% males, age: 64.3 ± 8.8, FEV_1_% of predicted: 48.9 ± 20) completed the pulmonary rehabilitation program. Very good responders had significantly worse baseline characteristics with a higher burden of disease, a higher proportion of rollator-users, higher body mass index (BMI), more limitations of activities in daily life, emotional dysfunction, higher symptoms of dyspnea and worse quality of life. Of the seven pre-identified clusters, ‘the overall best functioning cluster’ and ‘the low disease burden cluster’ both including the best 6MWD, the lowest dyspnea score and the overall best health status, demonstrated attenuated outcomes, while in ‘the cluster of disabled patients’, 76% of the patients improved health status with at least 2 times MCID. This ‘cluster of disabled patients’ as well as ‘the multimorbid cluster’, ‘the emotionally dysfunctioning cluster’, ‘the overall worst-functioning cluster’ and ‘the physically dysfunctioning cluster’ all demonstrated improvements in performance and satisfaction for occupational activities (more than 65% of patients improved with > 1MCID), emotional functioning (more than 50% of patients improved with > 1 MCID) and overall health status (more than 58%).

**Conclusion:**

The current study confirms the differential response to pulmonary rehabilitation based on multidimensional response profiling. Cluster analysis of baseline traits illustrates that non-linear, clinically important differences can be achieved in the most functionally and emotionally impaired clusters and that ‘the overall best functional cluster’ as well as ‘the low disease burden cluster’ had an attenuated outcome.

## Introduction

Pulmonary rehabilitation (PR) as an integrated, personalized intervention to improve the physical and psychological condition of patients with COPD, is based on a thorough assessment in order to identify treatable traits [1]. This concept of identification of pulmonary and extrapulmonary treatable traits has been proposed to apply personalized medicine to each individual and to improve outcomes by recognition of the individual needs [2]. In practice however, PR programs mostly consist of limited components based on a minimum set of identifiable traits [3]. Furthermore, current evidence for PR is based on changes in exercise performance and health status while the combination of interventions reflected in a set of multidimensional outcomes, is poorly addressed [4]. A previous study demonstrated that responses in regular outcomes are differential between patients and distinct multidimensional response profiles could be identified [4]. However, identification of the right patient for the right program as well as prediction of outcomes remains difficult [4]. Furthermore, different types of exercise-based care require an optimal profiling of patients with COPD. Recently, an expert-opinion model for referral to exercise-based care has been proposed based on disease instability, burden of disease, physical capacity and activity, irrespective of the widely applied degree of airflow limitation [5].

Clusters of COPD patients have been reported in order to individualize the treatment programs [6, 7]. Neither co-morbidity clusters, nor integrated respiratory physiomics clusters contributed to a better prediction of outcomes and to development of cluster based intervention strategies [8, 9]. Based on a thoroughly assessed set of pulmonary and extra-pulmonary traits at the start of a PR program, we recently described seven clusters of COPD patients [10].

The aims of this study are to confirm the differential response to pulmonary rehabilitation based on a previously reported set of response indicators in COPD patients and to assess the potential of previously identified traits-based clusters in order to predict these differential responses for future design of multidimensional and patient-centered interventions.

## Material and methods

### Study design

The current analysis is based on the data from the Chance Study: an observational, prospective, single-center study about COPD, health status and cardiovascular comorbidities in relation to the outcomes of PR [11]. This study was approved by the Medical Ethical Committee of the Maastricht University Medical Centre+ (METC 11-3-070) and is registered at http://www.trialregister.nl (NTR 3416). All patients gave written informed consent. The baseline results have been described previously [10].

### Study sample

COPD patients referred by chest physicians for a comprehensive PR program at Ciro (Horn, the Netherlands) were eligible to participate (See S1 File). Ciro is a specialized PR center in the southern part of the Netherlands, for patients suffering from complex underlying respiratory diseases [12].

### Interdisciplinary PR program

Ciro provides a state-of-the-art interdisciplinary PR program [13] for patients with COPD consisting of 40 sessions. An integrated 2.5-day pre-rehabilitation assessment, assessing physical, emotional and social traits, formed the basis for an individualized PR program [14]. PR can be inpatient (8 weeks, 5 days/week) or outpatient (8 weeks, 3 half days/week, followed by 8 weeks 2 half days/week). Patients were allocated for an outpatient or inpatient setting based on an interdisciplinary evaluation after assessment. In general, only care-dependent patients requiring extensive medical supervision were allocated to an inpatient PR program. The outpatient PR program took place under supervision of Ciro in 6 hospitals in the South East of the Netherlands. At the start and during the program, treatment goals were discussed in partnership with each patient. Interventions included physical exercise training, occupational therapy, nutritional counseling, psychosocial counseling, education and exacerbation management. Physical exercise training consists of strengthening exercises, treadmill walking and stationary cycling. Training intensity was monitored and scheduled at moderate-to-high intensity. Moreover, the training intensity increased during the rehabilitation period, based on dyspnea and fatigue symptom scores. All patients underwent general physical exercise for lower and upper extremities, and daily supervised 30-min outdoor walks. Patients, who were too dyspnoeic to perform endurance/interval/resistance training, received lower-limb high-frequency neuromuscular electrical stimulation [15]. Each individualized program was followed by an outcome measurement by trained technicians, who were not involved in the exercise training program. Reasons for drop-outs were not systematically scored but were largely related to interfering exacerbations, requiring hospitalization.

### Measurements

As described previously [7, 10], the pre-rehabilitation assessment includes the identification of pulmonary and extra-pulmonary (functional, behavioral and health status) attributes. For a detailed description see S2 File.

Changes in the degree of dyspnea were measured using the modified Medical Research Council (mMRC) scale (from grade 0 = no troubles with breathlessness to grade 4 = too breathless to leave the house) [16]. The COPD-specific version of the St George′s Respiratory Questionnaire (SGRQ-C), ranging from 0 (optimal) to 100 points (worst) evaluated changes in health status [17]. Exercise performance was measured by a 6-min walk test (six-minute walk distance, 6MWD, change in meters, m) [18] and a constant work-rate test (CWRT, change in cycle time expressed in seconds, s) [19], performed on a stationary bicycle at 75% of the pre-determined peak work rate (Carefusion, Houten, the Netherlands). The Canadian Occupational Performance Measure (COPM) was used to identify specific problematic activities of daily life. Patients scored how well they were performing the problematic activities of daily life (performance score; COPM-P) and how satisfied they were with this level of performance (satisfaction score; COPM-S) [20]. Scores range between 1 (“not able to do it” or “not at all satisfied”, respectively) to 10 points (“able to do it extremely well” or “extremely satisfied”). Symptoms of anxiety and depression were measured by the Hospital Anxiety and Depression Scale (HADS) with a total score ranging from 0 (optimal) to 21 (worst) points. A score of 11 points or higher indicates a severe mood disturbance [21]. All outcomes were compared with baseline assessment data.

### Statistics

All statistical analyses were performed using Viscovery SOMine 7.3 build 7427 by Viscovery Software GmbH (www.viscovery.net; Vienna, Austria). Self-organizing maps (SOMs, also referred to as Kohonen maps) were used to create an ordered representation of selected attributes. The SOM method can be viewed as a non-parametric regression technique that simplifies complexity by converting multidimensional data spaces into lower dimensional abstractions. A SOM generates a non-linear representation of the data distribution and allows the user to identify homogeneous data groups visually to reveal meaningful relationships. Using the topology of the created SOM model, clusters have been generated by applying the SOM-Ward Cluster algorithm of Viscovery, a hybrid algorithm that employs the classical hierarchical method of Ward on top of the SOM topology. When creating a SOM, no replacement of missing values is necessary, since only existing values are used to find the best matching position for each patient.

**Fig 1:**
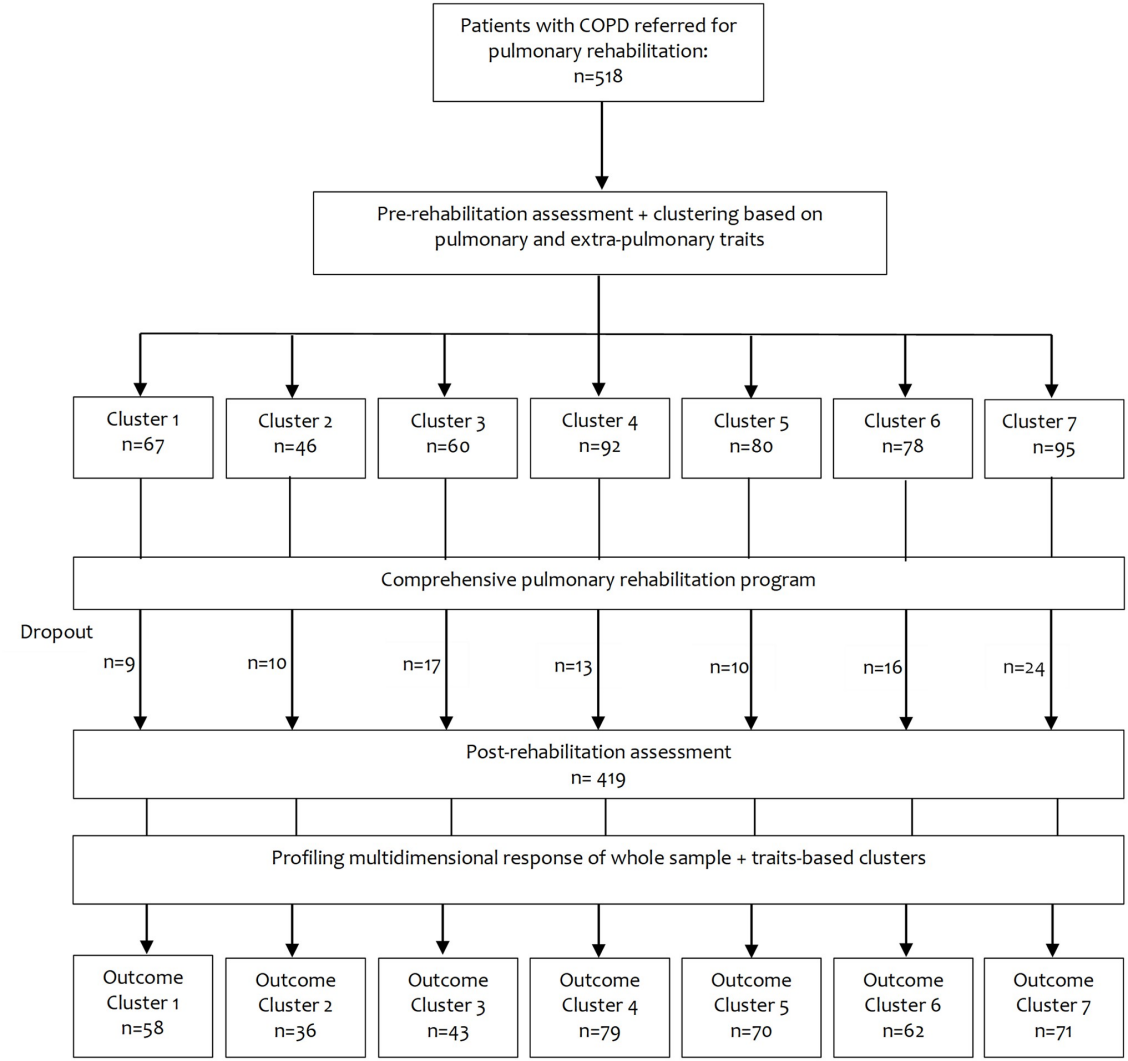
Patients before and after comprehensive pulmonary rehabilitation program.

**Fig 2:**
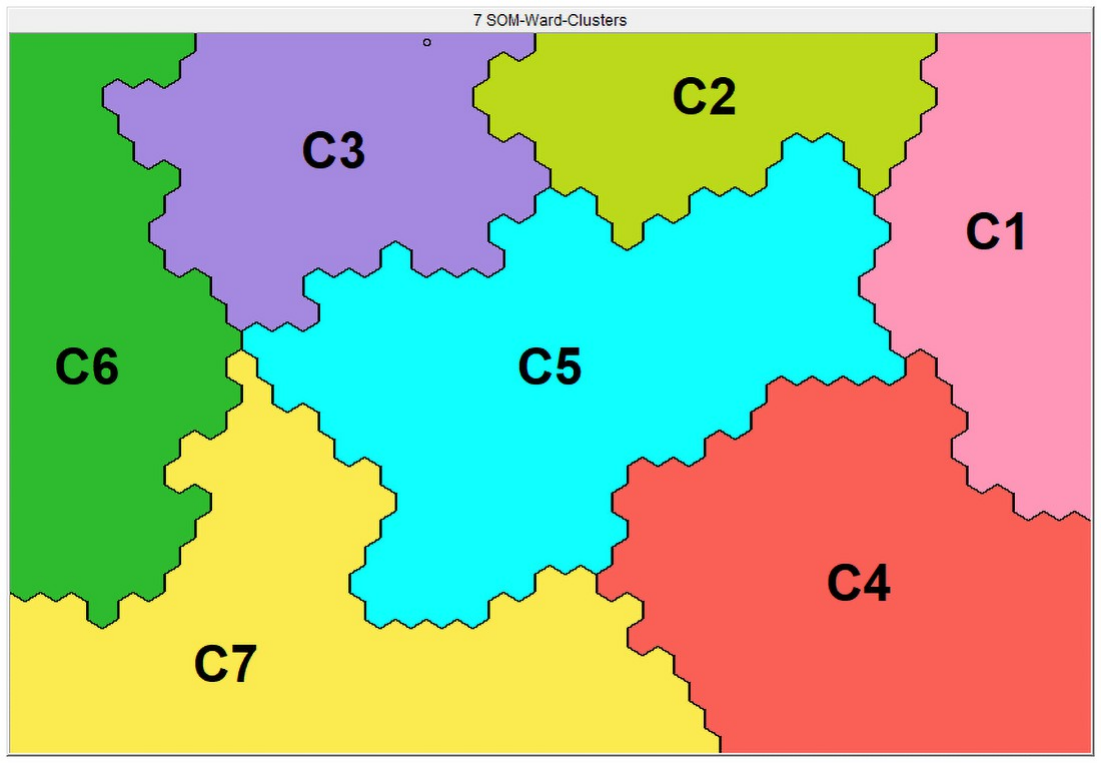
Seven different clusters. The Viscovery SOMine program placed all patients with COPD on a specific position on the map based on their baseline characteristics. Subjects located close to each other on the map resemble in terms of their baseline characteristics. Based on the SOM model created, seven SOM-Ward clusters with significantly different profiles have been generated: C1, ‘the overall best functioning cluster’; C2, ‘the ADL-limited cluster’; C3, ‘the multi-morbid cluster’; C4, ‘the low burden cluster’; C5, ‘the emotionally dysfunctioning cluster’; C6, ‘the overall worst functioning cluster’ and C7, ‘the physically dysfunctioning cluster’.

Based on the overall similarity concerning the pre-rehabilitation assessment, seven clusters could be identified (see Figs 1 and 2). A detailed description of these clusters was previously reported [10]. The seven clusters were described as: Cluster 1, ‘the overall best functioning cluster’; Cluster 2, ‘the ADL most limited cluster’; Cluster 3, ‘the multi- morbid cluster’; Cluster 4, ‘the low burden cluster’; Cluster 5, ‘the emotionally dysfunctioning cluster’; Cluster 6, ‘the overall worst functioning’ and Cluster 7, ‘the physically dysfunctioning cluster’.

The efficacy of the PR program of the whole sample and after traits-based clustering was evaluated based on the minimal clinically important difference (MCID) [4] for the following eight response indicators: 6MWD (+ 30 m); CWRT (+ 100 s); COPM-P (+ 2 points); COPM-S (+ 2 points); HADS-A (- 1,5 points); HADS-D (- 1,5 points); MRC dyspnea (−1 grade); and SGRQ-Total (- 4 points). The aforementioned response indicators were used to calculate the overall multidimensional response [4] in which indicators were weighted as follows: 6MWD: 28%; cycle endurance time CWRT: 20%; COPM-P: 6.5%; COPM-S 6.5%; HADS-A: 8%; HADS-D: 8%; MRC dyspnoea: 8%; and SGRQ-Total: 15%, summing up to 100%. The weights are chosen to be the same as used in Spruit et al. [4], which were based on estimations of importance of each indicator (i.e. indicators that are widely used to evaluate the effectiveness of PR were given more weight) as well as on the percentage of missing values of the respective indicator. The higher the percentage of missing values was, the lesser weight was given.

Based on this multidimensional response profiling different groups were generated with substantially different response profiles (very good responders, good responders, moderate responders, and poor responders). Baseline characteristics between these response groups were compared using the integrated two-sided T-test with a confidence level of 99%.

In the same way, responses to pulmonary rehabilitation were compared based on the pre-identified seven clusters based on baseline pulmonary and extra-pulmonary traits.

## Results

### Patient characteristics of the whole sample

518 COPD patients were included (see Table 1). They represent COPD patients with mild to very severe airflow limitation, a substantial smoking history, one or more comorbidities, an impaired diffusion capacity and elevated static lung volumes. On average, patients experienced a high impact of the disease on activities in daily life, an impaired health status, had an impaired physical performance and deconditioned quadriceps muscles. 24.1% of the patients used long term oxygen therapy (LTOT). The mean number of exacerbations as well as hospitalizations in the last year was on average 2.2 and 0.9. Accordingly the majority of the patients were therefore classified in GOLD B (26.2%) and D (54.8%).

**Table 1:** Demographics, clinical characteristics, pulmonary, extra-pulmonary, behavioral and health status of the whole sample, patients completing and not completing pulmonary rehabilitation.

	Whole sample	Patients completing pulmonary rehabilitation	Patients not completing pulmonary rehabilitation
Patients, n (%)	518 (100)	419 (81)	99 (19)
**Demographics and clinical characteristics**			
Women, %	44.4	44.6	43.4
Age, years	64.1 (9.1)	64.3 (8.8)	63.2 (10.3)
Smoking pack years	42.4 (23.6)	42 (23.6)	44.1 (23.5)
Exacerbations <1 year, n	2.2 (1.8)	2.2 (1.8)	2.3 (1.7)
Hospitalizations <1 year, n	0.9 (1.3)	0.8 (1.2)	1.1 (1.4)
Patients with LTOT use, %	24.1	24.8	21.2
Number of respiratory medications, n	7.2 (3.6)	7.1 (3.5)	7.2 (3.7)
Number of different kind of medications, n	6.8 (3.2)	6.8 (3.2)	6.8 (3.4)
Patients with GOLD I / II /III / IV, %	7.3 / 35.7 / 36.9 / 20.1	7.6 / 36.3 / 35.3 / 20.8	6.1 / 33.3 / 43.4 / 17.2
Patients with GOLD A / B / C / D, %	10.3 / 26.2 / 8.2 / 54.8	10.6 / 28.1 / 8.6 / 52.0	9.1 / 18.2 / 6.1 / 66.3
**Pulmonary traits**			
FEV_1_, % predicted	48.6 (20)	48.9 (20)	47.3 (20.1)
FVC, % predicted	97.5 (21.5)	98.5 (20.8)	92.9 (23.7)
FEV_1_/FVC, %	37.5 (12.2)	37.3 (12.1)	38.4 (12.9)
PEF, % of predicted	64.4 (24)	64.6 (23.7)	63.6 (25.2)
ITGV, % predicted	148.7 (35.9)	148.6 (36.3)	148.8 (34)
RV, % predicted	161.1 (50.7)	160.6 (50.9)	163.1 (49.7)
TLC, % predicted	117.1 (17.5)	117.5 (17.3)	115.4 (18.2)
TLCO, % predicted	49.3 (17.2)	50 (17.5)	46.7 (15.8)
KCO, % predicted	64 (21.9)	64.2 (21.9)	62.9 (22.1)
MIP, % predicted	78.5 (23.3)	79.9 (23.6)	72.8 (21) ^#^
MEP, % predicted	63.2 (20.4)	64.1 (20.6)	59.1 (19.2)
PaCO_2_, kPa	5.3 (0.9)	5.3 (0.9)	5.5 (0.9)
PaO_2_, kPa	9.5 (1.5)	9.6 (1.5)	9.5 (1.6)
SaO_2_, %	93.9 (3.2)	93.9 (3.1)	93.6 (3.7)
**Extra-pulmonary traits—physical**			
Quadriceps peak torque, % predicted	66.2 (18.9)	66.7 (18.9)	63.9 (19)
Six-MWD, m	424 (124.4)	431.1 (123.7)	393 (123.3) ^#^
Six-MWD, % predicted	67.1 (18)	68.5 (18)	61 (16.6) ^#^
Peak work rate, % predicted	55.5 (27.4)	55.9 (26.8)	53.9 (30)
Peak VO_2_, % predicted	66.2 (30.4)	66.6 (30)	64.2 (32.1)
CWRT, s	295.5 (218.7)	305.1 (225)	251.5 (181.5)
TUG, s	10.5 (3.4)	10.3 (2.8)	11.6 (5) ^#^
Use of rollator, %	28.5	27.3	33.3
BMI, kg/m^2^	26.2 (5.8)	26.2 (5.7)	26.2 (6.3)
FFMI, kg/m^2^	17.2 (2.6)	17.2 (2.6)	17.2 (2.7)
CCI, points	1.6 (1)	1.6 (0.9)	1.8 (1.3)
**Extra-pulmonary traits—behavioural and health status**			
CDS, points	69.4 (7.3)	69.7 (7.2)	68.4 (7.9)
COPM-P, points	3.9 (1.4)	3.9 (1.4)	3.7 (1.3)
COPM-S, points	3.3 (1.7)	3.4 (1.7)	3 (1.6)
HADS-A, points	7.8 (4.5)	7.5 (4.4)	9 (4.9) ^#^
HADS-D, points	7.5 (4.3)	7.4 (4.2)	8 (4.9)
mMRC dyspnea grade	2.4 (1)	2.4 (1)	2.7 (1)
CAT, total score, points	21.5 (6.6)	21.5 (6.6)	21.7 (6.9)
SGRQ, total score, points	61.1 (17.4)	60.1 (17.1)	65.4 (18.1) ^#^
CCQ, total score, points	2.6 (1)	2.6 (1)	2.8 (1.1)

Data are presented as mean (SD) or as a percentage of the whole sample.

*BMI*, body mass index; *CAT*, COPD assessment test; *CCI*, Charlson Comorbidity index; *CCQ*, Clinical COPD Questionnaire; *CDS*, Care Dependency Scale; *COPM-P*, Canadian Occupational Performance Measure-performance with; *COPM-S*, Canadian Occupational Performance Measure-satisfaction with; *CWRT*, constant work-rate test; *FEV*_*1*_, forced expiratory volume in 1 s; *FFMI*, fat-free mass index; *FVC*, forced vital capacity; *GOLD I*, *II*, *III*, *IV*, Global Initiative for Chronic Obstructive Lung Disease classification *I* (mild = FEV_1_ ≥ 80% predicted), *II* (moderate = 50% ≤ FEV_1_ < 80% predicted), *III* (severe = 30% ≤ FEV_1_ < 50%), *IV* (very severe = FEV_1_ < 30% predicted); *GOLD A*, *B*, *C*, *D*, Global Initiative for Chronic Obstructive Lung Disease classification *A* (mMRC 0–1, CAT < 10 and 0 or 1 exacerbation not leading to hospital admission), *B* (mMRC ≥ 2, CAT ≥ 10 and o or 1 exacerbation not leading to hospital admission), *C* (mMRC 0–1, CAT < 10 and ≥ 2 or ≥ 1 exacerbation leading to hospital admission), *D* (mMRC ≥ 2, CAT ≥ 10 and ≥ 2 or ≥ 1 exacerbation leading to hospital admission); *HADS-A*, Hospital Anxiety and Sepression Scale, anxiety scores; *HADS-D*, Hospital Anxiety and Depression Scale, depression scores; *ITGV*, intra thoracic gas volume; *KCO*, the single-breath transfer factor of the lung for carbon monoxide (TLCO) per unit alveolar volume; *LTOT*, long-term oxygen therapy; *MEP*, maximal static expiratory mouth pressure; *MIP*, maximal static inspiratory mouth pressure; *mMRC*, modified Medical Research Council; *PaCO*_*2*_, arterial partial pressure of carbon dioxide; *PaO*_*2*_, arterial partial pressure of oxygen; *PEF*, peak expiratory flow in 1 s; *RV*, residual volume; *SaO*_*2*_, arterial oxygen saturation; *SGRQ*, St. George’s Respiratory Questionnaire; *Six-MWD*, 6-minute walk distance; *TLC*, total lung capacity; *TUG*, Timed Up and Go test; *VO*_*2*_, oxygen uptake.

^#^: p<0.01 versus patients completing pulmonary rehabilitation.

Of the whole sample, drop out was 19% and 419 patients completed the PR program (see Fig 1 and Table 1). Of the patients not-completing PR, inspiratory mouth pressure, 6 MWD, Timed Up and Go test, anxiety and quality of life were significantly worse compared to the patients completing PR. However, all the other characteristics were comparable between completers and non-completers.

### Multidimensional response profiling of patients completing pulmonary rehabilitation

Table 2 summarizes the improvements following PR for the total group and after stratification for response. On average, improvements were found for 6MWD: 22.9 ± 67 m; CWRT: 206.4 ± 306 s; COPM-P: 2.8 ± 1.8 points; COPM-S: 3.5 ± 2.2 points; HADS-A: 1.7 ± 3.7 points; HADS-D: 2.1 ± 3.7 points; MRC: 0.3 ± 1.1 and SGRQ total score: 9.1 ± 14 points. A clinically important gain was achieved in 55.8 ± 27.8% of all outcomes. As expected, the very good responders group included the highest proportion of clinically relevant improvements: 82 ± 15.5% of outcomes exceeding more than 1 MCID and 62.7 ± 18.1% outcomes exceeding more than 2 MCID. Good responders showed 65 ± 16.1% outcomes exceeding more than 1 MCID and 37.5 ± 18.1% outcomes exceeding more than 2 MCID. While clinically relevant improvements were significantly lower in moderate and poor responders, as an example, 50.5% of the patients still improved more than 1 MCID for COPM-P and 59.6% for COPM-S in the group of moderate responders.

**Table 2:** Responses to pulmonary rehabilitation of all patients completing pulmonary rehabilitation.

	Patients completing PR	Very good responder	Good responder	Moderate responder	Poor responder
Patients, n (% patients completing PR)	419 (100)	108 (26)	146 (35)	123 (29)	42 (10)
Δ 6MWD, m	22.9 (67)	86.7 (66.2)	26.7 (31.9) ^#^	-9 (46) ^#^^,^^¶^	-65.9 (52.7) ^#^^,^^¶^^,^^+^
· ≥ 30 m, % patients	43.7	87.5	46.5^#^	16 ^#^^,^^¶^	0 ^#^^,^^¶^^,^^+^
· ≥ 60 m, % patients	21.8	62.5	14.8 ^#^	1.7 ^#^^,^^¶^	0 ^#^
Δ CWRT, s	206.4 (306)	442.8 (325)	237.7 (266.5) ^#^	61.9 (171.3) ^#^^,^^¶^	-96.1 (197.8) ^#^^,^^¶^^,^^+^
· ≥ 100 s, % patients	51.9	82.5	60.6 ^#^	31.6 ^#^^,^^¶^	0 ^#^^,^^¶^^,^^+^
· ≥ 200 s, % patients	36.2	75.3	37.9 ^#^	12.3 ^#^^,^^¶^	0 ^#^^,^^¶^
Δ COPM-P, points	2.8 (1.8)	4 (1.7)	3.2 (1.6) ^#^	1.8 (1.3) ^#^^,^^¶^	0.7 (1.3) ^#^^,^^¶^^,^^+^
· ≥ 2 points, % patients	68.3	86.5	80.6	50.5 ^#^^,^^¶^	22.9 ^#^^,^^¶^^,^^+^
· ≥ 4 points, % patients	26.2	54.8	27.6 ^#^	5.5 ^#^^,^^¶^	0 ^#^^,^^¶^
Δ COPM-S, points	3.5 (2.2)	4.7 (1.9)	4 (1.8) ^#^	2.5 (1.9) ^#^^,^^¶^	0.8 (1.5) ^#^^,^^¶^^,^^+^
· ≥ 2 points, % patients	76.6	93.3	90.3	59.6 ^#^^,^^¶^	26.5 ^#^^,^^¶^^,^^+^
· ≥ 4 points, % patients	43.3	63.5	51.5	26.6 ^#^^,^^¶^	2.9 ^#^^,^^¶^^,^^+^
Δ HADS-A, points	1.7 (3.7)	3.7 (3.7)	2.2 (3.2) ^#^	0.2 (3.3) ^#^^,^^¶^	-0.9 (2.9) ^#^^,^^¶^
· ≥ 1.5 points, % patients	50.5	71.9	54.9 ^#^	36.5 ^#^^,^^¶^	19.4 ^#^^,^^¶^
· ≥ 3.0 points, % patients	39.3	62.5	39.9 ^#^	27.1 ^#^	11.1 ^#^^,^^¶^
Δ HADS-D, points	2.1 (3.7)	3.9 (3.5)	3 (3.5)	0.5 (3.1) ^#^^,^^¶^	-0.7 (3) ^#^^,^^¶^
· ≥ 1.5 points, % patients	53	69.8	62.4	37.4 ^#^^,^^¶^	19.4 ^#^^,^^¶^
· ≥ 3.0 points, % patients	38.7	57.3	47.4	23.4 ^#^^,^^¶^	2.8 ^#^^,^^¶^^,^^+^
Δ mMRC dyspnea, grade	0.3 (1.1)	1 (1.1)	0.3 (1) ^#^	-0.1 (1) ^#^	-0.2 (0.8) ^#^
· ≥ 1 grade, % patients	38.9	67.6	38.7 ^#^	23 ^#^	13.8 ^#^
· ≥ 2 grades, % patients	15.6	33.8	14 ^#^	6.9 ^#^	0 ^#^
Δ SGRQ total score, points	9.1 (14)	19.2 (11.7)	12.2 (12.7) ^#^	1.7 (10.5) ^#^^,^^¶^	-4.9 (9.3) ^#^^,^^¶^^,^^+^
· ≥ 4 points, % patients	61.6	89.7	76.3 ^#^	36 ^#^^,^^¶^	15.4 ^#^^,^^¶^
· ≥ 8 points, % patients	50.9	83.5	63.7 ^#^	24.6 ^#^^,^^¶^	2.6 ^#^^,^^¶^^,^^+^
Outcomes exceeding ≥ 1 MCID, %	55.8 (27.8)	82 (15.5)	65 (16.1) ^#^	36.4 (18.7) ^#^^,^^¶^	13.4 (15.1) ^#^^,^^¶^^,^^+^
Outcomes exceeding ≥ 2 MCID, %	34.2 (25.9)	62.7 (18.1)	37.5 (18.1) ^#^	16.2 (14.1) ^#^^,^^¶^	2.3 (6.1) ^#^^,^^¶^^,^^+^

See legend Table 1 for explanation of abbrevations. Data are presented as mean (SD), unless otherwise stated. Δ, improvement (a minus sign means a deterioration); a lower score for HADS, mMRC dyspnea and SGRQ is an improvement. Outcomes exceeding ≥ x MCID, %: Percentage of outcomes which exceed the pre-defined minimal clinically important difference (MCID) at least x times.

^#^: p<0.01 versus very good responder cluster;

^¶^: p<0,01 versus good responder cluster;

^+^: p<0.01 versus moderate responder cluster.

When the value is significantly higher versus all the other clusters, the table-cell is colored red; if it is significantly lower, it is colored blue.

### Baseline characteristics after stratification for multidimensional response clusters

Table 3 summarizes the baseline characteristics of the very good responders, good, moderate and poor responders. Compared to the other groups, very good responders had significantly worse characteristics with a higher burden of disease, a higher proportion of rollator-users, higher BMI, higher limitations of activities in daily life, emotional dysfunction, higher symptoms of dyspnea and worse quality of life, while moderate responders demonstrated less hospitalizations, less limitations of activities in daily life, lower symptoms of dyspnea and a higher quality of life. Very good responders showed significantly better pulmonary traits compared to the other groups. The proportion of patients following an inpatient program was significantly higher in the very good responders compared to good, moderate and poor responders.

**Table 3:** Demographics, clinical characteristics, pulmonary, extra-pulmonary, behavioral and health status of very good, good, moderate and poor responders.

	Very good responder	Good responder	Moderate responder	Poor responder
Patients, n (% patients completing PR)	108 (26)	146 (35)	123 (29)	42 (10)
**Demographics and clinical characteristics**				
Women, %	43.5	46.6	43.1	45.2
Age, years	63.6 (8.8)	63.8 (9.2)	64.8 (8.3)	66.7 (8.9)
Smoking pack years	42.7 (21.1)	40.2 (18.9)	44.4 (30.4)	39.4 (21.1)
Exacerbations <1 year, n	2.9 (1.9)	2.1 (1.9) ^#^	1.9 (1.6) ^#^	1.9 (1.6) ^#^
Hospitalizations <1 year, n	1 (1.2)	0.9 (1.4)	0.6 (1) ^#^	1 (1.3)
Patients with LTOT use, %	27.8	25.3	23.6	19.1
Number of respiratory medications, n	8 (3.6)	7.1 (3.8)	6.6 (3.2) ^#^	6.5 (3)
Number of different kind of medications, n	7.7 (3.4)	6.7 (3.3)	6.4 (3) ^#^	6.3 (2.9)
Patients with GOLD I / II /III / IV, %	9.3 / 39.8 / 33.3 / 17.6	6.9 / 35.6 / 32.9 / 24.7	7.3 / 32.5 / 39 / 21.1	7.1 / 40.5 / 38.1 / 14.3
Patients with GOLD A / B / C / D, %	4.6 / 21.3 / 9.3 / 64.8	10.4 / 35.4 / 6.2 / 46.9 ^#^	16.4 ^#^ / 25.4 / 9.8 / 48 ^#^	9.5 / 28.6 / 12.2 / 48.8
**Pulmonary traits**				
FEV_1_, % predicted	50.6 (19.9)	47.6 (20.2)	48.2 (19.4)	50.7 (21.4)
FVC, % predicted	94.8 (21.2)	99.6 (21.5)	100.3 (19.3)	99.3 (21.2)
FEV_1_/FVC, %	40.3 (12.8)	35.8 (11.5) ^#^	36.1 (11.5) ^#^	38.2 (12.7)
PEF, % of predicted	69.6 (24.9)	63.7 (22.8)	61.4 (22.5) ^#^	64 (26)
ITGV, % predicted	139.6 (37.8)	151 (32.8)	152.2 (35.6)	150.7 (44)
RV, % predicted	152.1 (52.9)	165.5 (45.3)	161.7 (52)	160.4 (60.8)
TLC, % predicted	113.5 (18.4)	119.3 (16.2) ^#^	118.6 (16.2)	117.8 (20.6)
TLCO, % predicted	54.7 (17.2)	49 (18)	47.3 (16) ^#^	49.8 (19)
KCO, % predicted	72.2 (24.3)	61.3 (19.7) ^#^	61.4 (20.3) ^#^	64 (23.7)
MIP, % predicted	81.5 (20.5)	79.4 (25)	81.2 (23.5)	73.3 (26.1)
MEP, % predicted	64.9 (20.8)	65.6 (21.5)	62.9 (19)	61 (21.5)
PaCO_2_, kPa	5.4 (1)	5.3 (0.9)	5.2 (0.8)	5.2 (0.8)
PaO_2_, kPa	9.6 (1.7)	9.6 (1.4)	9.5 (1.4)	9.4 (1.5)
SaO_2_, %	93.8 (3.7)	94.1 (2.6)	94 (2.9)	93.6 (3.3)
**Extra-pulmonary traits—physical**				
Quadriceps peak torque, % predicted	69.4 (18.9)	65.3 (17.6)	67 (18.8)	64.4 (23)
6MWD, m	404.8 (140.2)	432 (121.1)	455.9 (104.8) ^#^	422.9 (128.5)
6MWD, % predicted	66.3 (19.8)	68.2 (18.1)	70.9 (15.4)	67.7 (19.5)
Peak work rate, % predicted	55.7 (27.1)	54.9 (27.6)	57.5 (27)	54.8 (22.6)
Peak VO_2_, % predicted	70.2 (30.7)	65.2 (30.9)	65.1 (28.1)	67.5 (31.1)
CWRT, s	318.4 (222.6)	286.8 (216.4)	304.8 (215.5)	335.8 (283.7)
TUG, s	10.8 (3.1)	10.4 (3)	9.7 (2.2) ^#^	10.4 (2.9)
Use of rollator, %	37.4	28.1	19.7 ^#^	21.4
BMI, kg/m^2^	28.7 (6.3)	26.3 (5.5) ^#^	24.2 (4.8) ^#^^,^^¶^	25.7 (4.8) ^#^
FFMI, kg/m^2^	18.2 (2.9)	16.9 (2.4) ^#^	16.7 (2.3) ^#^	16.8 (2.5) ^#^
CCI, points	1.7 (1)	1.5 (0.8)	1.6 (0.9)	1.9 (1.2)
**Extra-pulmonary traits—behavioural and health status**				
CDS, points	69 (7.6)	69.3 (6.8)	70.8 (5.9)	69.3 (10.3)
COPM-P, points	3.5 (1.2)	3.8 (1.4)	4.2 (1.3) ^#^	4.5 (1.6) ^#^
COPM-S, points	2.9 (1.6)	3.2 (1.6)	3.8 (1.7) ^#^^,^^¶^	4.1 (1.8) ^#^^,^^¶^
HADS-A, points	8.7 (4.4)	7.9 (4.3)	6.4 (4.2) ^#^^,^^¶^	6.8 (4.1)
HADS-D, points	8.7 (4.1)	8 (4.1)	6.1 (4) ^#^^,^^¶^	6.2 (3.6) ^#^
mMRC dyspnea grade	2.6 (1.1)	2.4 (1)	2.1 (1) ^#^^,^^¶^	2.3 (1.1)
CAT, total score, points	23.7 (6.3)	22.6 (5.9)	19 (6.3) ^#^^,^^¶^	19.5 (7.4) ^#^^,^^¶^
SGRQ, total score, points	67.5 (15)	61 (16.1) ^#^	53.8 (16.6) ^#^^,^^¶^	56.9 (19.6) ^#^
CCQ, total score, points	2.9 (1)	2.8 (1)	2.2 (0.8) ^#^^,^^¶^	2.4 (1.1)
**Inpatient %**	76	60 ^#^	41 ^#^^,^^¶^	38 ^#^

See legend Table 1 for explanation of abbreviations.

^#^, p<0.01 versus very good responder cluster;

^¶^, p<0,01 versus good responder cluster;

^+^, p<0.01 versus moderate responder cluster.

When the value is significantly higher versus all the other clusters, the table-cell is colored red; if it is significantly lower, it is colored blue.

### Responses to PR after traits-based clustering

The responses to PR for the seven traits-based clusters are summarized in Table 4.

**Table 4:** Responses to pulmonary rehabilitation for the seven clusters.

	Cluster 1 *The overall best functioning cluster*	Cluster 2: *The ADL-limited cluster*	Cluster 3: *The multi-morbid cluster*	Cluster 4: *he low burden cluster*	Cluster 5: *The emotionally dysfunctioning cluster*	Cluster 6: *The overall worst functioning cluster*	Cluster 7: *The physically dysfunctioning cluster*
Patients, n	67	46	60	92	80	78	95
Drop out, % patients	13.4	21.7	28.3	14.1	12.5	20.5	25.3
Patients completing PR, n	58	36	43	79	70	62	71
Inpatient, % %	18.2 ^#^^,^^¶^^,^^^^^,^^†^^,^^‡^	69.8 ^+^^,^^†^	74.6 ^+^^,^^†^	19.6 ^^^^,^^†^^,^^‡^	62 ^†^	92.2 ^‡^	70.2
6MWD, m	553.1 (71.9) ^#^^,^^¶^^,^^+^^,^^^^^,^^†^^,^^‡^	400.1 (86.4) ^¶^^,^^+^^,^^^^^,^^†^	344.1 (85.5) ^+^^,^^^^^,^^†^^,^^‡^	506.5 (83) ^^^^,^^†^^,^^‡^	465.7 (84.6) ^†^^,^^‡^	270.5 (103.8) ^‡^	403.5 (88.1)
Δ 6MWD, m	24.4 (51.5)	51.3 (87.3) ^+^^,^^‡^	25.1 (63.9)	5.5 (45.9) ^^^^,^^†^	28.4 (54.3)	46.3 (94.1) ^‡^	2.4 (65.3)
• ≥ 30 m, % patients	45.6	55.9 ^+^	53.5 ^+^	29.1 ^†^	49.3	56.6 ^‡^	30.9
• ≥ 60 m, % patients	19.3	38.2 ^+^^,^^‡^	23.3	10.1 ^†^	24.6	39.6 ^‡^	11.8
CWRT, s	453.8 (237.5) ^#^^,^^¶^^,^^^^^,^^†^^,^^‡^	259 (142.9)	213.9 (136.4) ^+^^,^^^^	352.3 (265.7) ^†^^,^^‡^	310.4 (206.9) ^†^^,^^‡^	211.9 (204.4)	231.2 (158.9)
Δ CWRT, s	295.5 (336.2) ^¶^^,^^‡^	227.2 (216.2)	116.7 (205.8)	220 (328.8)	264.9 (369.1)	157.4 (261.6)	130 (261.3)
• ≥ 100 s, % patients	69.2 ^¶^^,^^†^^,^^‡^	60	41.5	52	62.3 ^‡^	39.5	35.9
• ≥ 200 s, % patients	48.2 ^¶^	46.7	22	36	42	32.6	26.6
COPM-P, points	4.3 (1.4) ^#^^,^^†^	3.1 (1.1) ^¶^^,^^+^^,^^^^^,^^‡^	4.2 (1) ^+^^,^^†^	4.8 (1.2) ^^^^,^^†^^,^^‡^	3.9 (1.4) ^†^	2.7 (1) ^‡^	3.8 (1.2)
Δ COPM-P, points	2.9 (2.1)	3.6 (1.9) ^+^	2.7 (1.4) ^+^	1.7 (1.6) ^^^^,^^†^^,^^‡^	3 (1.9)	3.3 (1.8)	2.8 (1.5)
• ≥ 2 points, % patients	69.2	82.4 ^+^	74.4	49.3 ^†^^,^^‡^	66.2	74.1	74.6
• ≥ 4 points, % patients	28.9 ^+^	44.1 ^+^^,^^‡^	20.5	9.9 ^^^^,^^†^	33.9	37.9	17.5
COPM-S, points	3.9 (1.9) ^#^^,^^^^^,^^†^	2.6 (1.5) ^¶^^,^^+^	3.7 (1.2) ^†^	4.3 (1.6) ^^^^,^^†^^,^^‡^	3 (1.6)	2.5 (1.2) ^‡^	3.1 (1.6)
Δ COPM-S, points	3.5 (2.3)	4.1 (2.1) ^+^	3.2 (1.8)	2.6 (2) ^^^^,^^†^^,^^‡^	3.9 (2.3)	3.7 (2.5)	3.6 (1.8)
• ≥ 2 points, % patients	73.1	85.3	79.5	66.2	76.9	79.3	82.3
• ≥ 4 points, % patients	38.5	52.9	41	28.2 ^^^^,^^†^	50.8	53.5	43.6
HADS-Anxiety, points	6.4 (3.6) ^#^^,^^+^^,^^^^^,^^†^	9.1 (3.9) ^+^^,^^†^^,^^‡^	7.9 (4.1) ^+^^,^^†^	4.6 (2.9) ^^^^,^^†^^,^^‡^	9.6 (4) ^†^^,^^‡^	11.7 (4.4) ^‡^	6.5 (4.2)
Δ HADS-A, points	1.8 (3.5)	1.6 (3.7)	1.6 (3.6)	0.3 (3.1) ^^^^,^^†^^,^^‡^	2.6 (3.6)	2.7 (4.6)	1.7 (3.3)
• ≥ 1.5 points, % patients	47.2	51.5	55.9	36.5 ^^^^,^^†^	61.7	62.3	46.2
• ≥ 3.0 points, % patients	37.7	36.4	44.1	21.6 ^^^^,^^†^	50	50.9	40
HADS-Depression, points	5.9 (3.7) ^#^^,^^¶^^,^^+^^,^^^^^,^^†^	9.5 (4.5) ^+^^,^^‡^	8.3 (3.5) ^+^^,^^†^	4 (2.5) ^^^^,^^†^^,^^‡^	8.9 (3.9) ^†^^,^^‡^	11.2 (3.3) ^‡^	6.7 (4.3)
Δ HADS-D, points	1.3 (3.8) ^†^	2.9 (4.4) ^+^	2.4 (3) ^+^	0.9 (2.7) ^^^^,^^†^	3.1 (3.6)	3.8 (4.1) ^‡^	1.5 (3.5)
• ≥ 1.5 points, % patients	39.6 ^^^^,^^†^	57.6 ^+^	55.9	31.1 ^^^^,^^†^^,^^‡^	73.3	67.9	53.9
• ≥ 3.0 points, % patients	35.9	42.4	38.2	25.7 ^†^	46.7	56.6 ^‡^	32.3
mMRC dyspnea, grade	1.3 (0.8) ^#^^,^^¶^^,^^^^^,^^†^^,^^‡^	2.7 (0.9) ^+^^,^^^^^,^^†^	3.1 (0.7) ^+^^,^^^^^,^^†^^,^^‡^	1.6 (0.7) ^^^^,^^†^^,^^‡^	2.2 (0.8) ^†^^,^^‡^	3.5 (0.7) ^‡^	2.7 (0.8)
Δ mMRC dyspnea, grade	-0.2 (1) ^#^^,^^¶^^,^^†^	0.9 (1.5) ^+^	0.8 (1.1) ^+^	0 (0.7) ^†^	0.3 (1) ^†^	0.8 (0.9) ^‡^	0.2 (1.1)
• ≥ 1 grade, % patients	23.9 ^#^^,^^¶^^,^^†^	53.9 ^+^	60 ^+^	19.3 ^†^	36.4 ^†^	66.7 ^‡^	36.4
• ≥ 2 grades, % patients	4.4 ^#^^,^^¶^^,^^†^	38.5 ^+^	33.3 ^+^	1.8 ^†^	13.6	25	13.6
SGRQ total, points	43.3 (15.1) ^#^^,^^¶^^,^^^^^,^^†^^,^^‡^	71.6 (9.3) ^+^^,^^^^^,^^†^^,^^‡^	66.2 (12.5) ^+^^,^^†^	43.8 (11.6) ^^^^,^^†^^,^^‡^	67 (8.2) ^†^	77.9 (10) ^‡^	63.6 (15.3)
Δ SGRQ total score, points	9.1 (17.4)	15.8 (13) ^+^	9.1 (11.5) ^+^	2 (13.3) ^^^^,^^†^^,^^‡^	11.1 (10)	10.5 (13)	10.9 (14.9)
• ≥ 4 points, % patients	64.2 ^+^	81.8 ^+^	59	38.7 ^^^^,^^†^^,^^‡^	74.6	63.6	62.7
• ≥ 8 points, % patients	52.8 ^+^	75.8 ^+^	53.9 ^+^	25.3 ^^^^,^^†^^,^^‡^	60.3	58.2	49.3
Outcomes exceeding ≥1 MCID, %	54.2 (29.1) ^+^	66.7 (24.6) ^+^^,^^‡^	59.7 (28.7) ^+^	40.5 (26.6) ^^^^,^^†^^,^^‡^	63.3 (25.5)	62.6 (28)	53.1 (24.1)
Outcomes exceeding ≥2 MCID, %	33 (25.2) ^+^	47.2 (27.5) ^+^^,^^‡^	33.8 (22.8) ^+^	20.2 (20.7) ^^^^,^^†^^,^^‡^	40.7 (24.7) ^‡^	43.9 (30) ^‡^	29.6 (21.6)
Very good responders, % patients	29.3 ^+^	52.8 ^+^^,^^‡^	25.6 ^+^	7.6 ^^^^,^^†^	28.6	38.7 ^‡^	15.5
Good responders, % patients	29.3	33.3	37.2	32.9	42.9	33.9	33.8
Moderate responders, % patients	32.8	5.6 ^+^	23.3	45.6 ^^^^,^^†^	21.4	19.4 ^‡^	40.9
Poor responders, % patients	8.6	8.3	14	13.9	7.1	8.1	9.9

See legend Table 1 for explanation of abbreviations. Data are presented as mean (SD), unless otherwise stated. Δ, improvement (a minus sign means a deterioration); a lower score for HADS, mMRC dyspnea and SGRQ is an improvement. Outcomes exceeding ≥ x MCID, %: Percentage of outcomes which exceed the pre-defined minimal clinically important difference (MCID) at least x times.

^#^, p<0.01 versus cluster 2;

^¶^, p<0,01 versus cluster 3;

^+^, p<0.01 versus cluster 4;

^^^, p<0.01 versus cluster 5;

^†^, p<0.01 versus cluster 6;

^‡^, p<0.01 versus cluster 7.

When the value is significantly higher versus all the other clusters, the table-cell is colored red; if it is significantly lower, it is colored blue.

In *Cluster 1*, *the overall best functioning cluster* at baseline, drop-out was 13.4% with 58 patients completing the PR program. The proportion of patients following an inpatient program was 18.2%. Except for constant work rate test, which improved significantly after treatment, response indicators in cluster 1 were comparable to the average of the response indicators of the whole sample. Breathlessness even worsened significantly after PR in this cluster.

Despite the higher impairment in ADL, higher depression scores and worse quality of life in *cluster 2*, this *ADL limited cluster* (drop out 21.7%, 36 patients of which 69.8% inpatients) manifested a significantly higher proportion of very good responders compared to the other clusters. In addition, the percentage of patients having outcomes exceeding ≥ 2 MCID was similar to cluster 6 and significantly higher than the other clusters. A significant better response to PR was found for 6 MWD, performance of problematic activities of daily life, symptoms of dyspnea and health status.

*Cluster 3*, *the more multimorbid cluster* (drop out 28.3%, 43 patients, 74.6% inpatients) with significantly lower values for exercise performance and higher dyspnea scores at baseline showed a response pattern comparable to the whole sample with exception for dyspnea which improved significantly better in this cluster.

*Cluster 4* (drop out 14.1%, 79 patients, 19.6% inpatients), identified as *the low burden cluster* had similar baseline characteristics as cluster 1, but demonstrated the lowest proportion of very good responders, the highest proportion of poor responders, lowest percentage of outcomes exceeding ≥ 1 MCID and lowest percentage of outcomes exceeding ≥ 2 MCID. Only improvement for CWRT was similar to the entire group. All other indicators responded worse compared to the other clusters.

*Cluster 5*, *the emotionally dysfunctioning cluster* (drop out 12.5%, 70 patients, 62% inpatients) demonstrated at baseline a significantly better 6MWD, but these patients had higher scores for anxiety and depression and worse health status compared to all patients. Although the majority of the response indicators in cluster 5 were comparable to the whole group, PR particularly resulted in an improvement of the burden of depression.

*Cluster 6* (drop out 20.5%, 62 patients, 92.2% inpatients) was identified as *the worst functioning cluster*. However, PR resulted in a higher proportion of outcomes with a clinical important difference as well as a higher percentage of very good responders. In particular, a better response was found for 6 MWD, performance of problematic activities of daily life, symptoms of depression and symptoms of dyspnea.

*Cluster 7*, *the physically dysfunctioning cluster*, (drop out 25.3%, 71 patients, 70.2% inpatients), showed smallest improvement in physical functioning parameters after PR while other response indicators were comparable to the mean of the whole sample.

**Fig 3:**
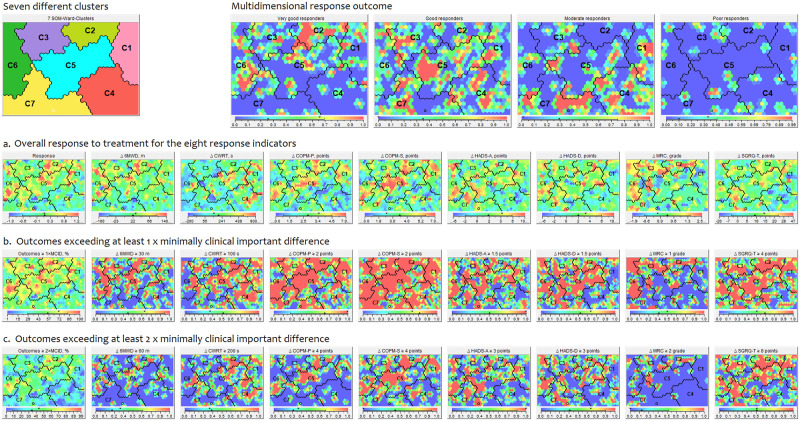
Outcomes after pulmonary rehabilitation for the seven traits-based clusters. Panels generated using Viscovery (Viscovery Software GmbH, Vienna, Austria). The seven different traits-based clusters are demonstrated in the upper left corner. The four panels next to it, illustrate (in red) the proportion of very good responders, good responders, moderate responders and poor responders for the different clusters.

The overall response as well as the outcomes, expressed in absolute terms as well as in changes of MCIDs, is illustrated in Fig 3. Fig 3 clearly illustrates the differential response after PR in COPD, but also that poor responders form only a minority when multidimensional response profiling is conducted and that the different response profiles are distributed 0ver all clusters. The individual components contributing to the multidimensional profile are depicted in Fig 3b. To note is the distribution of the outcomes in performance and satisfaction in performing activities of daily life and the improvement in psychological burden as well as the improvements in experienced health status over the identified clusters at the end of the PR program.

When looking at the first panel of a) patients “raise a red flag” if they had a very good response, “a green flag” when the response was good to moderate, and “a blue flag” when the response was poor. In this way maps can be interpreted. Refer to the color scale below each attribute picture to match colors with attribute values. All other attribute pictures in a) are the absolute change in Medical Research Council (MRC) dyspnoea grade, 6-min walk distance (6MWD), cycle endurance time (constant work-rate test; CWRT), Canadian Occupational Performance Measure, performance (COPM-P), Canadian Occupational Performance Measure, satisfaction (COPM-S), Hospital Anxiety and Depression Scale, anxiety (HADS-A), Hospital Anxiety and Depression Scale, depression (HADS-D), and St. George’s Respiratory Questionnaire total score (SGRQ-T). b) Left panel shows the proportion of clinically relevant outcomes (exceeding at least 1× minimally clinical important difference (MCID)). All other panels are the proportion of patients per outcome showing a clinically relevant improvement (exceeding at least 1× MCID). c) First panel shows the proportion of clinically relevant outcomes (exceeding at least 2× MCID). All other panels are the proportion of patients per outcome showing a clinically relevant improvement (exceeding at least 2× MCID).

## Discussion

The present study confirms the tremendous heterogeneity in pulmonary and extra-pulmonary trait impairments in COPD patients referred for PR. The current results corroborate that the response to PR is differential in patients with COPD, which justifies the use of a multidimensional outcome to assess the efficacy of comprehensive PR programs. Furthermore, our study confirms that the differential response to such PR program can be clustered based on multidimensional performance metrics, including evaluation of the functional and emotional daily life disease burden, in identification of groups of patients with very good, good, moderate or poor response. Impaired physical capacity and high disease burden despite better lung function identify the very good responding patients. Clustering based on identified traits may help to identify PR candidates: particularly those patients with low disease burden or overall best functioning are less prone to benefit from PR.

Starting from the first authoritative statement on PR, an accurate diagnosis of the physiopathological and psychopathological manifestations of pulmonary diseases has been put forward [22]. In the latest definition, PR has been described as a comprehensive, individualized intervention based on a thorough patient assessment [13]. More recently, in order to realize a personalized or precision medicine approach, the concept of treatable traits has been introduced [2, 23, 24]. To cope with these traits, the organization of PR as a flexible, individualized and integrated intervention, based on partnering different skills has been described [14]. To reflect the complexity and heterogeneity of COPD, we recently described that baseline traits could be grouped into 7 discernible clusters [10]. Current practice for PR referral is in sharp contrast with this personalized approach and largely ignores this complexity and heterogeneity in impairments. Indeed, the degree of airflow limitation is still predominantly used as criterium to select patients for PR [25, 26] despite the overwhelming evidence that airflow limitation is a very poor predictor of exercise capacity, physical activity or burden of the disease [27]. Even clustering of integrated respiratory physiomic characteristics does not allow adequate prediction of PR outcomes [9]. In the most recent Global Initiative for Obstructive Lung Diseases (GOLD) strategy document, PR is just encouraged for those patients with high symptom burden and at risk for exacerbations [28]. GOLD recommends a formal rehabilitation program that takes into account the individual characteristics of a COPD patient, however, these characteristics are not further specified.

Our study confirms previous findings that based on a carefully selected set of key performance measures and validated values for MCID, a differential response to PR can be demonstrated varying from very good responders to poor responders [4]. Previous studies reported that individual patients respond differentially on various types of outcomes [29, 30]. In the group of disabled COPD patients of the current study more than 50% improved health status with at least 2 times the MCID while most evidence-based pharmacological interventions showed marginally clinical relevant differences [31]. Very good responders demonstrated the highest disease burden, manifested by lower exercise performance, worse scores on activities in daily living, anxiety, depression and quality of life although they had better pulmonary traits.

Our data even suggest the role of the current intervention in the reduction of anxiety and depression [32]. Previous data reported that coping styles and/or changes in coping styles are related to changes in anxiety and depression after PR and that good responders particularly decreased their passive coping style [33]. At least our data illustrate that in the majority of the patients PR improves the physical, emotional and social functioning of the patient [34]. This study confirms previous findings showing that sex, age, the degree of airflow limitation or even ambulatory oxygen therapy cannot be used to predict outcomes of PR [4]. Although not systematically explored, it seems that patients in the cluster with a higher percentage of drop-out, largely related to interfering exacerbations, were even more impaired in physical capacity and disease burden than the other clusters.

Although in- and outpatient programs are matched in terms of the composition and scheduled interventions, the number of very good and good responders is remarkably higher after in-patient rehabilitation. In-patient rehabilitation was based on the interdisciplinary evaluation of care dependency and needs for extensive medical supervision. We previously reported the need for more holistic, personalized approaches to optimize the patient’s quality of life integrating the patient’s whole environment and the team of health care professionals in co-creating value care [35]. It is now well recognized that patient’s personalities, health beliefs, social support networks, financial resources and other unique life circumstances have important effects how an individual patient respond to treatment [35]. These context- and program- based influences have been largely neglected in previous selection criteria for pulmonary rehabilitation settings in COPD patients [36]. Further studies are needed to explore this co-creating value in health caring of inpatient rehabilitation [37].

Identification of patients or clusters of patients that do or do not respond to PR will be an important step to improve the cost-effectiveness of PR. Based on a thorough assessment of a broad trait panel and applying a multidimensional set of key performance measures, this study aimed to evaluate the outcome predictability of previously identified clusters of COPD [10]. At least 2 clusters with attenuated response to PR could be identified, the so-called overall best functioning cluster and the cluster with overall low disease burden. Remarkable are the effects of pulmonary rehabilitation in the overall worst functioning and the physically dysfunctioning cluster, clusters of disabled patients: 58 and 49% of these patients improved health status with at least 2 times the minimally clinically defined difference. Despite these cluster differences, our data underscores the need for a personalized trait profile and checking individual management goals at the start of a PR program.

Furthermore, while PR is generally advocated as a standard of care to improve shortness of breath, health status and exercise tolerance [38], our data suggest a shift towards performance and satisfaction of daily life activities as well as towards attenuation of the emotional and overall burden of the disease [4, 25]. Indeed, current guidelines focus on symptom and risk reduction as PR outcomes in line with outcomes of pharmacological interventions, while by definition PR has broader aims [13]. Therefore, it will become important to realize that for COPD patients as well as for chronic patients in general a more dynamic vision on outcomes will be needed considering health as a state of wellbeing characterized by the physical, mental and social potential [39, 40]. PR as comprehensive management must address the needs of the patients by evidence-based and efficient interventions in combination with health caring in response with the feelings that matter for the patient [37]. Besides physical training, PR aims to create continuous healing relationships, customized according the patients’ needs and values and in partnership with the patient [41]. In this way PR has the potential to improve resilience in the different health domains as well as the individual’s well-being [39, 40]. This approach conducted in specialized PR programmes is completely different from exercise-based care programmes, generally described also as PR interventions.

The present results are observational and definitive conclusions need to be based on further prospective data. At least, the current results aim to contribute to repositioning of PR as a comprehensive, personalized intervention tackling the multiple physical, emotional and/or social treatable traits of the referred patient and to stimulate the discussions on real patient-related outcomes. Considering this complexity, the organization of pulmonary rehabilitation is more than scheduling trait based interventions, but needs to tackle this heterogenous burden of the disease [34]. The strength of this study is the in-depth assessment of a wide set of traits, the supervision of the whole rehabilitation program as well as the broad outcome evaluation by independent technicians. One of the weaknesses of the study is of course that the data are derived from a single center for PR, hampering the generalization of our findings, and that no follow-up data are available. Another potential limitation of this study could be that the PR program is conducted according the 2013 ATS/ERS recommendations on PR [13]. At that time, the scope of PR was more directed on symptom relief and on exercise intolerance in particular rather than on the emotional and social domains. Furthermore, the current multidimensional response profiling is based on a set of eight expert-opinion outcome measures, with allocating the greatest importance to physical performance. More concise patient-derived and–related outcomes need to be developed in the future to better describe the dynamic changes of health transition in these patients.

To conclude, the current study confirms the differential response to PR based on multidimensional response profiling and provides detailed insights in trait complexity and performance metrics in patients referred for PR. Cluster analysis of baseline traits illustrates that clinically important differences can be achieved in the most functionally frail and emotionally impaired clusters and that the overall best functional as well as the low burden cluster manifested an attenuated outcome. The time has come to start up the discussion how pulmonary rehabilitation will be re-organised not to reduce impairments or symptoms but to improve the functional, emotional and social domains of health in patients with COPD and other chronic respiratory conditions.

## Supporting information

S1 File Eligibility criteria for chance study. (DOCX)

S2 File Measurements and statistics of the previously identified clusters. (DOCX)
