# Supplementary material for: Multidimensional outcome assessment of pulmonary rehabilitation in traits-based clusters of COPD patients
Source: PLoS One. 2022 Feb 17;17(2):e0263657. doi: 10.1371/journal.pone.0263657 (PMC8853536; doi:10.1371/journal.pone.0263657)
Supplement: S1 File — (DOCX) [file pone.0263657.s001.docx]

**S1 File: Eligibility criteria for Chance study**

Eligibility criteria are described in Smid, D.E., et al., *Impact of cardiovascular comorbidities on COPD Assessment Test (CAT) and its responsiveness to pulmonary rehabilitation in patients with moderate to very severe COPD: protocol of the Chance study.* BMJ Open, 2015. **5**(7): p. e007536. [1]

Patients were recruited by a specialized pulmonary rehabilitation centre. The inclusion of subjects started in April 2012. The inclusion of the subjects from the tertiary care setting has been completed mid-2014. These were patients with COPD referred for clinical assessment and pulmonary rehabilitation to Ciro, Horn, the Netherlands.

Patients were eligible if they fulfilled the following criteria:

1. Age 40-85 years.
2. A diagnosis of COPD according to GOLD guidelines.
3. Referral for assessment and pulmonary rehabilitation in Ciro by a chest physician.

*Exclusion criteria for the patients with COPD:*

1. A history of asthma, lung cancer, sarcoidosis, tuberculosis, lung fibrosis, cystic fibrosis or any other significant respiratory disease.
2. A moderate or severe exacerbation or pneumonia requiring systemic corticosteroids, antibiotics or hospitalisation during the last 4 weeks.
3. Having undergone lung surgery (e.g. lung volume reduction, lung transplantation).
4. Any clinically relevant disease which in the opinion of the investigator may influence the results of the study.
5. Malignancy within the last 5 years.
6. For primary care patients: treatment by respiratory physician in secondary or tertiary care.

For secondary care patients: treatment in tertiary care setting in the previous 5 years.

1. Smid, D.E., et al., *Impact of cardiovascular comorbidities on COPD Assessment Test (CAT) and its responsiveness to pulmonary rehabilitation in patients with moderate to very severe COPD: protocol of the Chance study.* BMJ Open, 2015. **5**(7): p. e007536.
