# Supplementary material for: Multidimensional outcome assessment of pulmonary rehabilitation in traits-based clusters of COPD patients
Source: PLoS One. 2022 Feb 17;17(2):e0263657. doi: 10.1371/journal.pone.0263657 (PMC8853536; doi:10.1371/journal.pone.0263657)
Supplement: S2 File — (DOCX) [file pone.0263657.s002.docx]

**S2 Appendix:**

**Measurements and statistics of the previously identified clusters**

Clusters are described in detail in Augustin, I.M.L., et al., *Incorporating Comprehensive Assessment Parameters to Better Characterize and Plan Rehabilitation for Persons with Chronic Obstructive Pulmonary Disease.* J Am Med Dir Assoc, 2020 [1].

**Measurements**

As described previously [1, 2], the pre-rehabilitation assessment includes the identification of pulmonary and extra-pulmonary (functional, behavioral and health status) attributes.

Pulmonary traits were determined by a comprehensive lung function assessment and existed of airflow limitation, static hyperinflation, gas transfer, respiratory pressures and arterial blood gases. Post-bronchodilator spirometry was performed to assess forced expiratory volume in 1 second (FEV_1_) and forced vital capacity (FVC). Spirometry was measured with Masterlab® (Jaeger, Würzburg, Germany) following ATS/ERS guidelines [3]. Values are expressed as percentage of predicted according the Global Lung Function Initiative [4]. Total lung capacity (TLC), residual volume (RV) and intra thoracic gas volume (ITGV) were determined through body-plethysmography (Masterlab® Jaeger, Würzburg, Germany) following the quality control guidelines [5]. Values are expressed as a percentage of the European Coal and Steel Community predicted values [6]. TLCO was measured following the standard of the single-breath determination of carbon monoxide [7] and expressed in the reference values of Cotes and colleagues [8]. Additionally, TLCO per unit alveolar volume (KCO) was calculated. Maximal static inspiratory (MIP) and expiratory mouth pressures (MEP) were assessed according to ATS/ERS guidelines [9] and expressed in the reference values according to Black and Hyatt [10]. Resting arterial partial pressure of oxygen (PaO_2_), carbon dioxide (PaCO_2_) and oxygen saturation were measured (GEM4000, Instrumentation Laboratory, Peachtree City, USA). Patients with long term oxygen therapy (LTOT) continued oxygen supply during the procedure. All lung function measurements were performed by certified and experienced respiratory technicians.

Measured functional extra-pulmonary traits (physical) were muscle strength, physical functioning, body composition and presence of comorbidities. Isokinetic quadriceps peak torque was measured using a Biodex (Biodex Medical Systems, Inc., New York, USA) [11, 12]. Exercise performance was assessed by a 6 minute walk test (6MWT) performed according to international guidelines [13]. The longest distance covered in two separate tests was registered as the 6 minute walk distance (6MWD). Furthermore, a symptom-limited incremental cardiopulmonary exercise test (CPET) was performed using an electrically, braked cycle ergometer (Carefusion, Houten, the Netherlands) including the measurement of maximal oxygen uptake (Peak VO_2_, ml/min) and maximal work rate in Watts (Peak work rate) [14]. Endurance exercise capacity was measured by a sub-maximal exercise test at 75% of the peak work rate (CWRT) [15]. Body mass index (BMI) was calculated by body weight in kg / (height in m)^2^. Fat-free mass (FFM) was assessed using dual-energy X-ray absorptiometry (DEXA) scan (Lunar Prodigy scan, GE Healthcare, Madison, WI, USA) and FFM (=lean mass + bone mineral content) was divided by squared height (in m) to obtain the FFM-index (FFMI) [16]. Bone mineral density (BMD by DEXA scan) was measured at the hip, lumbar spine, and whole body [17]. Self-reported comorbidities were assessed using the Charlson Comorbidity Index (CCI) [18].

Extra-pulmonary behavioral traits reflected by social functioning and emotional function, symptom perception and health status were assessed. The Care Dependency Scale (CDS), consisting of 15 items regarding basic and instrumental activities of daily living, was used to assess the level of care dependency [19]. The Canadian Occupational Performance Measure (COPM) was used to identify specific problematic activities of daily life [20]. Patients scored how well they were performing the problematic activities of daily life (performance score; COPM-P) and how satisfied they were with this level of performance (satisfaction score; COPM-S). Timed Up and Go (TUG) test was assessed to measure functional mobility [21]. Emotional functioning has been measured using the Hospital Anxiety and Depression Scale (HADS) [22], which is divided into an anxiety subscale (HADS-A) and a depression subscale (HADS-D). The degree of dyspnea was measured using the modified Medical Research Council (mMRC) scale [23]. The disease-specific health status was assessed using the COPD Assessment Test (CAT) [24], a simple questionnaire to measure the impact of COPD on a person’s life, and using the COPD-specific version of the St George's Respiratory Questionnaire (SGRQ-C) [25] that consists of three domains scores (symptoms, activity and impact) and a total score. Furthermore, the Clinical COPD Questionnaire (CCQ), a self-administered questionnaire consisting of three domains specially developed to measure clinical control in patients with COPD, was used [26].

In addition the assessment included demographics, medical history, the number of exacerbations and hospitalizations for COPD in the last twelve months, and the use of long-term oxygen (LTOT). Patients with COPD were classified as Global Initiative for Chronic Obstructive Lung Disease (GOLD) I (mild = FEV_1_ ≥ 80% predicted), II (moderate = 50% ≤ FEV_1_ < 80% predicted), III (severe = 30% ≤ FEV_1_ < 50%), IV (very severe = FEV_1_ < 30% predicted) and GOLD A (mMRC 0-1, CAT < 10 and 0 or 1 exacerbation not leading to hospital admission), B (mMRC ≥ 2, CAT ≥ 10 and o or 1 exacerbation not leading to hospital admission), C (mMRC 0-1, CAT < 10 and ≥ 2 or ≥ 1 exacerbation leading to hospital admission), D (mMRC ≥ 2, CAT ≥ 10 and ≥ 2 or ≥ 1 exacerbation leading to hospital admission) [27].

**Statistics**

Self-organizing maps (SOMs, also referred to as Kohonen maps) were used to create an ordered representation of the selected attributes. The SOM method can be viewed as a non-parametric regression technique that simplifies complexity by converting multi-dimensional data spaces into lower dimensional abstractions. A SOM generates a non-linear representation of the data distribution and allows the user to identify homogeneous data groups visually to reveal meaningful relationships. All statistical analyses were performed using Viscovery SOMine 7.1 by Viscovery Software GmbH (<http://www.viscovery.net>, Vienna, Austria).

Patients were ordered by their overall similarity concerning the attributes of the pre-rehabilitation assessment, which are the following in descending order of priority: FEV_1_/FVC in %; FEV_1_, FVC, PEF, ITGV, RV,TLC, TLCO, KCO, MIP and MEP (all in % predicted); mMRC dyspnea grade; CAT, total score; SGRQ, total score; CCQ, total score; CDS, item daily activities in points and CDS, total points; COPM-P in points; 6MWD in m; Peak work rate in Watts; Quadriceps peak torque in % predicted; TUG test in s; FEV_1_ in L; Airway resistance effective measured during bodyplethysmography in KPa*sec/L; COPD GOLD I-IV; HADS-D in points; Users of rollator in %; HADS-A in points; CAT score section impact on activities; CDS, item mobility; 6MWD in % predicted; LTOT users in %; CDS, item eating and drinking in points; CDS, item getting dressed and undressed in points; Quadriceps muscle endurance (Total Work in Joules); SGRQ score, section activity in points; SGRQ score, for section impact in points; COPD related hospital admissions last 12 months; SGRQ score, section symptom in points; arterial blood gases (PaCO_2_, cHCO_3_, PaO_2_, SaO_2_) and CCQ scores for functional state; and to a small extent absolute measures of FVC; PEF; ITGV; RV; TLC; TLCO; KCO; TLC by helium dilution technique; inspiratory vital capacity (IC); breath holding time (TA); MIP; and MEP.

Based on the created SOM model, clusters were generated using the SOM-Ward Cluster algorithm of Viscovery, a hybrid algorithm that applies the classical hierarchical method of Ward on top of the SOM topology.

1. Augustin, I.M.L., et al., *Incorporating Comprehensive Assessment Parameters to Better Characterize and Plan Rehabilitation for Persons with Chronic Obstructive Pulmonary Disease.* J Am Med Dir Assoc, 2020.

2. Augustin, I.M.L., et al., *The respiratory physiome: Clustering based on a comprehensive lung function assessment in patients with COPD.* PLoS One, 2018. **13**(9): p. e0201593.

3. Miller, M.R., et al., *Standardisation of spirometry.* Eur Respir J, 2005. **26**(2): p. 319-38.

4. Quanjer, P.H., et al., *Multi-ethnic reference values for spirometry for the 3-95-yr age range: the global lung function 2012 equations.* Eur Respir J, 2012. **40**(6): p. 1324-43.

5. Coates, A.L., et al., *Measurement of lung volumes by plethysmography.* Eur Respir J, 1997. **10**(6): p. 1415-27.

6. Quanjer, P.H., et al., *Lung volumes and forced ventilatory flows.* Eur Respir J, 1993. **6 Suppl 16**: p. 5-40.

7. Macintyre, N., et al., *Standardisation of the single-breath determination of carbon monoxide uptake in the lung.* Eur Respir J, 2005. **26**(4): p. 720-35.

8. Cotes, J.E., et al., *Standardization of the measurement of transfer factor (diffusing capacity).* Eur Respir J, 1993. **6 Suppl 16**: p. 41-52.

9. American Thoracic Society/European Respiratory, S., *ATS/ERS Statement on respiratory muscle testing.* Am J Respir Crit Care Med, 2002. **166**(4): p. 518-624.

10. Black, L.F. and R.E. Hyatt, *Maximal respiratory pressures: normal values and relationship to age and sex.* Am Rev Respir Dis, 1969. **99**(5): p. 696-702.

11. Borges, O., *Isometric and isokinetic knee extension and flexion torque in men and women aged 20-70.* Scand J Rehabil Med, 1989. **21**(1): p. 45-53.

12. Ribeiro, F., et al., *Test-retest reliability of lower limb isokinetic endurance in COPD: A comparison of angular velocities.* Int J Chron Obstruct Pulmon Dis, 2015. **10**: p. 1163-72.

13. Holland, A.E., et al., *An official European Respiratory Society/American Thoracic Society technical standard: field walking tests in chronic respiratory disease.* Eur Respir J, 2014. **44**(6): p. 1428-46.

14. American Thoracic, S. and P. American College of Chest, *ATS/ACCP Statement on cardiopulmonary exercise testing.* Am J Respir Crit Care Med, 2003. **167**(2): p. 211-77.

15. van 't Hul, A., R. Gosselink, and G. Kwakkel, *Constant-load cycle endurance performance: test-retest reliability and validity in patients with COPD.* J Cardiopulm Rehabil, 2003. **23**(2): p. 143-50.

16. Schols, A.M., et al., *Nutritional assessment and therapy in COPD: a European Respiratory Society statement.* Eur Respir J, 2014. **44**(6): p. 1504-20.

17. Graat-Verboom, L., et al., *Whole-Body versus Local DXA-Scan for the Diagnosis of Osteoporosis in COPD Patients.* J Osteoporos, 2010. **2010**: p. 640878.

18. Charlson, M.E., et al., *A new method of classifying prognostic comorbidity in longitudinal studies: development and validation.* J Chronic Dis, 1987. **40**(5): p. 373-83.

19. Dijkstra, A., et al., *Further psychometric testing of the Dutch Care Dependency Scale on two different patient groups.* Int J Nurs Pract, 2002. **8**(6): p. 305-14.

20. Annegarn, J., et al., *Problematic activities of daily life are weakly associated with clinical characteristics in COPD.* J Am Med Dir Assoc, 2012. **13**(3): p. 284-90.

21. Mesquita, R., et al., *Within-day test-retest reliability of the Timed Up & Go test in patients with advanced chronic organ failure.* Arch Phys Med Rehabil, 2013. **94**(11): p. 2131-8.

22. Zigmond, A.S. and R.P. Snaith, *The hospital anxiety and depression scale.* Acta Psychiatr Scand, 1983. **67**(6): p. 361-70.

23. Mahler, D.A. and C.K. Wells, *Evaluation of clinical methods for rating dyspnea.* Chest, 1988. **93**(3): p. 580-6.

24. Jones, P.W., et al., *Development and first validation of the COPD Assessment Test.* Eur Respir J, 2009. **34**(3): p. 648-54.

25. Jones, P.W., F.H. Quirk, and C.M. Baveystock, *The St George's Respiratory Questionnaire.* Respir Med, 1991. **85 Suppl B**: p. 25-31; discussion 33-7.

26. van der Molen, T., et al., *Development, validity and responsiveness of the Clinical COPD Questionnaire.* Health Qual Life Outcomes, 2003. **1**: p. 13.

27. GOLD. *Global Strategy for Prevention, Diagnosis and management of COPD*. 2021.
